# Efficacy of anti-PD-1/PD-L1 antibodies after discontinuation due to adverse events in non-small cell lung cancer patients (HANSHIN 0316)

**DOI:** 10.1186/s12885-018-4819-2

**Published:** 2018-10-03

**Authors:** Motoko Tachihara, Shunichi Negoro, Takako Inoue, Motohiro Tamiya, Yuki Akazawa, Takeshi Uenami, Yoshiko Urata, Yoshihiro Hattori, Akito Hata, Nobuyuki Katakami, Soichiro Yokota

**Affiliations:** 1 Department of Respiratory Medicine, Kobe University Graduate School of Medicine, 7-5-1, Kusunoki-Cho, Chuo-Ku, Kobe-city, Hyogo 650-0017 Japan; 2 Department of Medical Oncology, Hyogo Cancer Center, 13-70 Kitaoji-cho, Akashi-shi, Hyogo 673-8558 Japan; 3 Department of Thoracic Oncology, Osaka International Cancer Institute, 3-1-69 Otemae, Chuo-Ku, Osaka-shi, Osaka, 541-8567 Japan; 4 Department of Thoracic Oncology, National Hospital Organization Toneyama National Hospital, 5-1-1 Toneyama, Toyonaka, Osaka, 560-8552 Japan; 5 Department of Thoracic Oncology, Hyogo Cancer Center, 13-70 Kitaoji-cho, Akashi-shi, Hyogo 673-8558 Japan; 6 Department of Medical Oncology, Kobe City Medical Center General Hospital, 2-1-1 2 Minatojima−Minamimachi, Chuo−ku, Kobe, 650−0047 Japan

**Keywords:** Anti-PD-1/L1 inhibitor, Non-small cell lung cancer, Adverse event, Discontinuation

## Abstract

**Background:**

Immune checkpoint inhibitors (ICIs) have emerged as promising therapeutic agents in non-small cell lung cancer (NSCLC). However, the duration for which ICIs should be continued remains a clinical problem.

**Methods:**

We examined the efficacy of anti-PD-1/PD-L1 inhibitors after the discontinuation of antibodies due to adverse events (AEs) in patients with NSCLC. This was a multicenter retrospective study that analyzed NSCLC patients who were treated with PD-1/PD-L1 inhibitors by August 2016.

**Results:**

The patients with NSCLC were 18 males and 1 female at a median 67 years of age (range: 49–80 years). Eighteen of 19 patients were treated with nivolumab, one was with atezolizumab. Approximately half of AEs were interstitial pneumonia. Fourteen patients (73.7%) were treated with steroid therapy. The median number of treatment cycles was 7 (range, 1–70), and the median duration of treatment was 2.8 months (range, 1 day-32.9 months). The overall response rate with confirmation during treatment was 21.1%. The median progression-free survival (PFS) was 10.2 months (95% confidence interval [CI] = 3.2–17.1 months) and 5.6 months (95% CI = 0–12.2 months) from the initiation and the discontinuation of PD-1/PD-L1 treatment, respectively. The median PFS after discontinuation according to the confirmed response during administration was not reached for partial response (PR) and 4.9 months (95% CI, 3.7–6.0) for stable disease (SD) patients (*P* = 0.02).

**Conclusion:**

The PFS of the PR patients was completely different from that of the SD patients. The cases with PR prior to the onset of AE tended to show a durable response after the discontinuation of PD-1/PD-L1 inhibitors.

**Electronic supplementary material:**

The online version of this article (10.1186/s12885-018-4819-2) contains supplementary material, which is available to authorized users.

## Background

Recent progress in the treatment of advanced non-small cell lung cancer (NSCLC) has been remarkable and promising. The development of immunotherapy resulted in a paradigm shift for the treatment of NSCLC. The PD-1 receptor is an immune checkpoint inhibitor expressed on activated T cells that downregulates excessive immune responses [1, 2]. Binding of PD-1 to its ligands on tumor cells suppresses T cells through negative feedback, leading to escape from the immune response [3–5]. Immune checkpoint inhibitors (ICIs), including anti-PD-1/PD-L1 antibodies, aim to restore antitumor immunity and have shown good clinical responses and an improved overall survival (OS) in patients with several tumors, such as melanoma and lung cancer [6–12]. In addition, ICIs notably show a durable clinical benefit persisting long after the cessation of therapy in several tumors [10–16].

Treatment with anti-PD-1/PD-L1 antibodies is associated with toxicities known as immune-related adverse events (irAEs) [6–17]. The typical median time from therapy initiation to irAEs was reported to be within 3 months, but the frequency of AEs increase with continued therapy [8, 13]. Thus, it is important to determine the appropriate period of administration of anti-PD-1/PD-L1 antibodies.

To estimate this period, we retrospectively evaluated the efficacy of PD-1/PD-L1 inhibitors after the discontinuation of these antibodies due to AEs in patients with NSCLC at Japanese cancer research institutes.

## Methods

### Patients and treatment

Patients with advanced NSCLC who received either single-agent anti-PD-1 or PD-L1 antibody until August 31, 2016, and stopped due to AEs at institutions participating in the Hanshin Cancer Research Group were included. Anti-PD-1 antibody, nivolumab was administered at 3 mg/kg every 2 weeks and anti-PD-L1 antibody, atezolizumab was at 1200 mg/body every 3 weeks. Patients’ medical records were retrospectively reviewed, and the following information was retrieved: age, gender, lung cancer histology, Eastern Cooperative Oncology Group (ECOG) performance status (PS), smoking status, treatment line, treatment for AEs, and number of treatment cycles. The histological classification of lung cancer was defined based on the World Health Organization pathology classification. Routine chest radiography was conducted every cycle to evaluate the treatment responses and AEs.

### Clinical analyses

Chest computed tomography (CT) was performed every 4 to 16 weeks as a routine procedure and were used to confirm disease response or progression. The treatment effect was evaluated with the Response Evaluation Criteria in Solid Tumors, version 1.1 (RECIST 1.1). Clinical response to therapy, the progression-free survival (PFS), and the OS were evaluated. This study was approved by the ethics committee of Kobe University and each institution.

### Statistical analyses

To analyze the OS and PFS, survival curves were plotted using the Kaplan-Meier method. The PFS with anti-PD-1/PD-L1 antibodies was assessed from the initiation of PD-1/PD-L1 treatment to the day on which the first objective signs of disease progression or death were recorded. The OS was calculated from the date of initiation of the anti–PD-1/PD-L1 treatment to the date of death and was censored at the date of last visit for patients whose deaths could not be confirmed. The survival probabilities between groups were compared using a log-rank test. All analyses were run PASW statistics version 18. A spider plot depicted the change from baseline for tumors for each subject in a study by week.

## Results

### Patients characteristics

Of the 192 patients who underwent anti-PD-1/PD-L1 treatment, we analyzed 19 patients (18 men and 1 woman) in whom treatment was stopped due to irAEs. The median length of follow-up was 16.9 months (range, 4.3–50.2 months). Patient characteristics are shown in Table 1 (Detail information of each patient was showed in Additional file 1: Table S1). Histology showed that 13 patients had squamous cell carcinoma, 4 patients had adenocarcinoma, 1 patient had adenosquamous cell carcinoma, and 1 patient had large-cell carcinoma. All patients had a history of smoking (45 pack-years, range 12.5–116 pack-years) and had a good PS (0 or 1). PD-1/PD-L1 inhibitors were administered as a second-line treatment for 6 patients, third-line for 8, and as fourth-line or later for 5. Eighteen of 19 patients were treated with nivolumab, one was with atezolizumab. The median number of administration was 7 times (1–70).

**Table 1:** Patient characteristics

Characteristics	number of patients (*n* = 19)
Gender	Male 18, Female 1
Median age (range), years	67 (49–80)
Histology	Squamous cell carcinoma 13 Adenocarcinoma 4 Adenosquamous cell carcinoma 1 Large cell carcinoma 1
Smoke history	Current 6, Former 13
Pack-years (range)	45 pack-years(12.5–116)
Performance status	PS0 1, PS1 18
Treatment line	2nd line, 6 3rd line, 8 More than 4th line, 5
PD-1/PD-L1 antibodies	Nivolumab 18 Atezolizumab 1
Adverse events	Interstitial pneumonia 10 diarrhea 2 hematological toxicity 2 anaphylactic 2, eruption 1, hemoptysis 1, hypophysitis1
Treatment for adverse events	Steroid 14, others 5

### Data related to adverse effects

The AEs were interstitial pneumonitis (*n* = 10), hematological toxicity (*n* = 2), enterocolitis (n = 1), diarrhea (n = 1), anaphylactic (n = 1), hypophysitis (n = 1), eruption (n = 1), and bleeding (n = 1). Fourteen patients (73.7%) were treated with corticosteroids for irAEs.

### Data related to survival

**Fig. 1:**
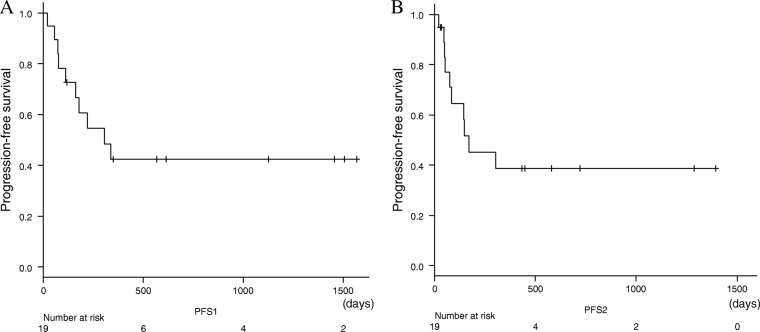
Kaplan-Meier curves of progression-free survival (PFS). **a** PFS from the treatment. **b** PFS from the discontinuation

**Fig. 2:**
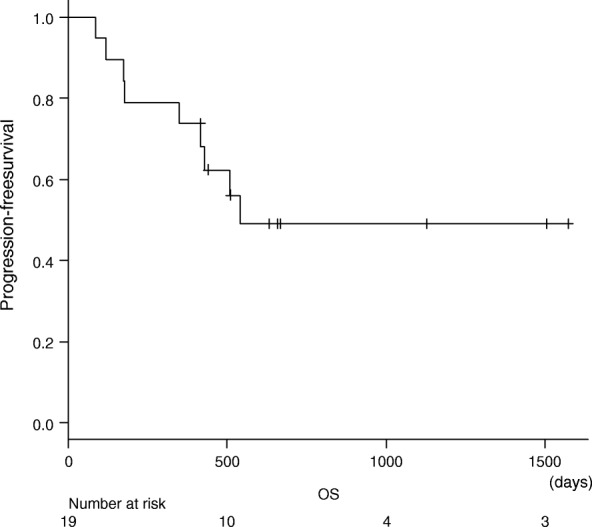
Kaplan-Meier curves of overall survival (OS)

**Fig. 3:**
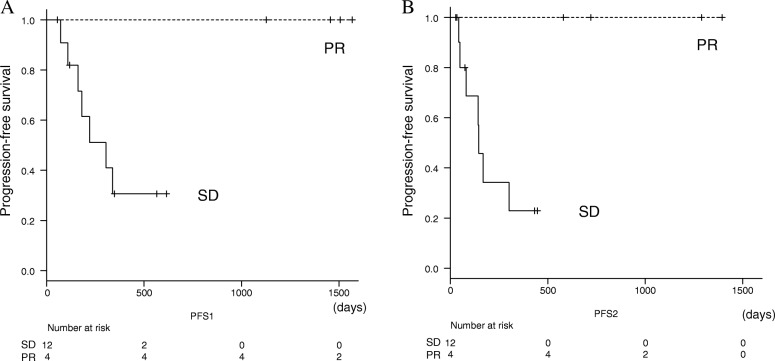
Kaplan-Meier curves of PFS according to the confirmed response during treatment. **a** PFS from the treatment. **b** PFS from the discontinuation

The confirmed overall response rate during treatment was 21.1% (4 of 19 patients) (Table 2). One partial response (PR) patient changed to complete response (CR), and 2 stable disease (SD) patients changed to 2 PR afterwards despite discontinuation of PD-1/PD-L1 inhibitors. The median PFS was 10.2 months (95% confidence interval [CI] = 3.2–17.1 months) and 5.6 months (95% CI = 0–12.2 months) from the initiation and the discontinuation of PD-1/PD-L1 treatment, respectively (Fig. 1). The median OS was 21.9 months (95% CI, 9.2–34.6) (Fig. 2). The median PFS according to the confirmed response during administration from treatment was not reached for PR patients and 10.2 months (95% CI, 3.9–16.4) for SD patients, respectively (*P* = 0.04) (Fig. 3a). The median PFS after discontinuation was not reached for PR and 4.9 months (95% CI, 3.7–6.0) for SD patients (*P* = 0.02) (Fig. 3b).

**Table 2:** Efficacy of PD-1/PD-L1 inhibitors

	*n* = 19
median number of Cycle (range)	7 (1–70)
Duration of treatment	2.8 months (1 day-32.9 months)
Best response during administration^a^	PR 4, SD 12, PD 1, NE 2
Best response including after discontinuation^a^	CR 1, PR 5, SD 11, PD 2 (PR → CR 1, SD → PR 2, NE → SD, PD1)

*CR* complete response, *PR* partial response, *SD* stable disease, *PD* progressive disease, *NE* not evaluated

^a^According to RECIST 1.1; Confirmed by a later scan performed at least 4 weeks after initial response was observed

**Fig. 4:**
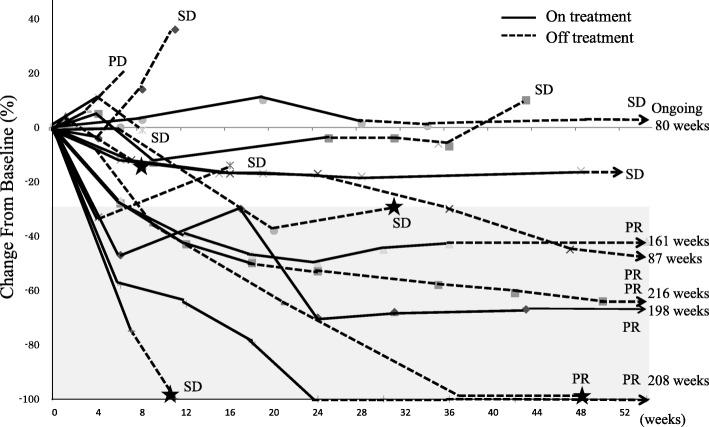
Spider plot. Tumor burden kinetics in patients with advanced non–small-cell lung cancer treated with PD-1/PD-L1 therapy. Baseline tumor measurements are standardized to zero. Tumor burden was measured as sum of longest diameters of target lesions compared with baseline. Percent change in target lesion tumor burden from baseline over time. Only includes patients with baseline target lesion and one or more post baseline target lesion assessments with no missing value (*n* = 16). Gray zone denote more than 30% decrease. Solid line and dotted line indicate on treatment and off treatment respectively. Star show occurrence of new lesion

The spider plot showed tumor burden kinetics in patients with NSCLC treated with PD-1/PD-L1 inhibitors (*n* = 16) (Fig. 4). The antitumor effect tended to plateau with 24-week administration of PD-1/PD-L1 inhibitors. In patients with SD at 24 weeks, a further antitumor effect was not achieved with or without the treatment, except for in 1 patient. Even in those with an antitumor effect, 2 of 4 cases that had stopped the treatment within 8 weeks showed aggravated disease with the appearances of new lesions afterwards. The other 2 cases showed a durable response (8–12 month) with the ultimate appearance of new lesions. The patients with PR at 12 weeks in whom the administration was continued for 12–24 weeks had good prognoses.

## Discussion

One of the major issues with ICIs is determining the treatment duration has the best balance of high efficacy and low toxicity. The present study evaluated the efficacy of anti-PD-1/PD-L1 antibodies after their discontinuation in patients with NSCLC and estimated the optimum period of treatment, considering risks and benefits. To our knowledge, this is the first study to investigate the duration for which anti-PD-1/PD-L1 antibodies should be continued. Two findings from the present study warrant mention. First, the prognoses in PR patients were completely different from those in SD patients. Second, the PR patients had good prognoses as long as the agents had been administered for a certain period. Our findings suggest that the appropriate period of prescription was 3 to 6 months in patients in whom AEs occurred.

Immunotherapy including anti-PD-1/PD-L1 antibodies has the potential for long-term disease control through the activation of the patients’ own immune system against cancer cells in several kinds of cancer [7–13]. The Kaplan-Meier curves of PFS showed that the slope of the curve flattened out after 6 months for patients treated with PD-1/PD-L1 antibodies [11, 14]. It has also become clear that the antitumor effect lasted even if the ICIs were stopped due to AEs or the prescribed treatment period expired [10–15]. In our study, the antitumor effect tended to fluctuate in the first 8–12 weeks and plateaued with 24-week administration of PD-1/PD-L1 inhibitors. Most of cases with SD at 8–12 weeks didn’t show PR, even if antibodies were continued afterward. Furthermore, 1 patient with SD at 24 weeks showed disease progression 20 weeks after stopping treatment. These data suggested that SD cases ought to be treated for as long as possible. This may explain why the PFS was completely different between cases with SD and PR.

In contrast, even if there was a good antitumor effect at first, most patients showed aggravation if treatment was stopped within 8 weeks. The patients with PR at 8–12 weeks in whom the administration was continued for 12–24 weeks at least seemed to have a good long-term response. Syukuya et al. reported that the landmark PFS correlated with the OS, with a longer landmark PFS at 24 weeks being the best predictor of the survival in patients with NSCLC treated with anti–PD-1/PD-L1 antibodies [16].

Recently, CheckMate 153 (phase IIIB/IV study) evaluated the clinical benefit of a fixed duration (1 year) of nivolumab treatment vs. continuous treatment in patients with previously treated advanced NSCLC as a secondary endpoint. The result showed that patients with continuous treatment of nivolumab for more than 1 year had a significantly better prognosis than those with fixed-duration treatment [17, 18]. However, while some cases showed a durable response in the discontinuation group, others showed aggravated disease afterwards despite continuous treatment. These data suggest that the immune response depends on the individual and cannot be stated unconditionally. Research for the acquired resistance mechanisms to ICI is also under way. Optimal use of ICI will hinge on the identification of mechanistic biomarkers of response, irAE, and resistance [19].

ICIs induce various types of irAEs, including late-onset irAEs after long-term prescription [17, 20–24]. Santini et al. reported retreatment with anti-PD-1/L1 therapy resulted in recurrent or new irAEs in half of patients with irAEs that had improved. Furthermore, among those with CR/PR prior to onset of irAEs, the PFS and OS were similar in the retreatment and discontinuation cohorts [25]. These present and previous findings suggest that patients who were apparent responders prior to the occurrence of AEs might not need retreatment. Given the risks and benefits, we must decide on whether or not to continue treatment on a case-by-case basis.

The present study has several limitations, such as its retrospective design and small population. Furthermore, we targeted patients who had been unable to avoid stopping treatment due to AEs, with no cases having been stopped at the patient’s wish at our institutions. The development of irAEs is reportedly associated with the survival outcome in patients with NSCLC receiving nivolumab treatment [26].

Since we focused on the patients who discontinued ICIs due to AEs, our result might apply only to select patients with a good prognosis. However, despite these limitations, our results may support suggestions to solve a major clinical problem associated with ICIs.

## Conclusion

The PFS of the PR patients was completely different from that of the SD in AE occurred patients. The cases with a disease status better than PR at the time of AE occurrence tended to show a durable response after the discontinuation of PD-1/PD-L1 inhibitors.

## Additional file

Additional file 1: Characteristics and clinical course of each patient. (XLSX 19 kb)

## Abbreviations

AEs: Adverse events
CR: Complete response
CT: Computed tomography
ECOG: Eastern Cooperative Oncology Group
ICIs: Immune checkpoint inhibitors
irAEs: immune-related adverse events
NSCLC: Non-small cell lung cancer
OS: Overall survival
PFS: Progression-free survival
PR: Partial response
PS: Performance status
RECIST 1.1: Response Evaluation Criteria in Solid Tumors
SD: Stable disease
